# On the Fatigue Performance of Friction-Stir Welded Aluminum Alloys

**DOI:** 10.3390/ma13194246

**Published:** 2020-09-23

**Authors:** Sergey Malopheyev, Igor Vysotskiy, Daria Zhemchuzhnikova, Sergey Mironov, Rustam Kaibyshev

**Affiliations:** Laboratory of Mechanical Properties of Nanoscale Materials and Superalloys, Belgorod National Research University, 308015 Belgorod, Russia; malofeev@bsu.edu.ru (S.M.); visotsky@bsu.edu.ru (I.V.); zhemchuzhnikova@bsu.edu.ru (D.Z.); rustam_kaibyshev@bsu.edu.ru (R.K.)

**Keywords:** aluminium alloys, friction-stir welding, fatigue

## Abstract

This work was undertaken in an attempt to ascertain the generic characteristics of fatigue behavior of friction-stir welded aluminum alloys. To this end, different alloy grades belonging to both the heat-treatable and non-heat-treatable types in both the cast and wrought conditions were studied. The analysis was based on the premise that the fatigue endurance of sound welds (in which internal flaws and surface quality are not the major issues) is governed by residual stress and microstructure. Considering the relatively low magnitude of the residual stresses but drastic grain refinement attributable to friction-stir welding, the fatigue performance at relatively low cyclic stress was deduced to be dictated by the microstructural factor. Accordingly, the fatigue crack typically nucleated in relatively coarse-grained base material zone; thus, the fatigue strength of the welded joints was comparable to that of the parent metal. At relatively high fatigue stress, the summary (i.e., the cyclic-plus residual-) stress may exceed the material yield strength; thus, the fatigue cracking should result from the preceding macro-scale plastic deformation. Accordingly, the fatigue failure should occur in the softest microstructural region; thus; the fatigue strength of the welded joint may be inferior to that of the original material.

## 1. Introduction

Due to the high stress concentrations and intrinsic defects, welded structures usually exhibit poor fatigue performance. This is particularly the case for aluminum welds, whose exceptionally low characteristics are widely known and even necessitate using a riveting approach for manufacturing of joint assemblies. However, a recent invention of an advanced friction-stir welding (FSW) technique may principally change this situation. Because of the solid-state nature of this welding process, the produced joints contain no solidification defects and are characterized by relatively low residual stress, as well as the fine-grained recrystallized microstructure [1,2]. 

Given significant potential of FSW for transportation industry, considerable research effects have been undertaken to explore fatigue behavior of aluminum friction-stir joints [3,4,5,6,7,8,9,10,11,12,13,14,15,16]. As expected, their fatigue properties are typically found to be superior to that of comparable fusion welds [3,4]. Based on the analysis of a large array of experimental data, it has been suggested that the fatigue life of the friction-stir welded (FSWed) aluminum is primarily governed by the following four factors: (a) welding defects, (b) surface quality, (c) residual stress, and (d) microstructure [2]. 

It is widely known that the volumetric defects associated with improper FSW conditions could essentially degrade fatigue strength. In addition to those, however, the so-called “kissing bond” defect [17] is also of a great concern. Due to a specific character of FSW process, a short segment of the unwelded butt surface normally remains at the weld root and may serve as a precursor for the fatigue crack nucleation [7,8,9]. This circumstance must be taken into account when considering even a nominally defect-free joint. 

In the light of the well-known initiation of the fatigue failure at the material surface, the characteristic tool marks, which are normally produced during FSW at the upper weld surface, may also represent a considerable problem. Due to the relative sharp profile, these marks may give rise to the stress concentrations during fatigue tests and thus promote the crack nucleation [2,10]. 

Similar to the conventional fusion welding, FSW results in local material heating. The concomitant thermal expansion of the hot material gives rise to the tensile residual stress in the weld zone; those are normally equilibrated by the compressive stresses generated in the base material section. It is well accepted that the tensile stress promotes the fatigue failure whereas the compression stress suppresses the fatigues cracks. Despite the magnitude of the FSW-induced residual stress is typically much lower than that generated during the conventional fusion welding, they, nevertheless, may also exert a considerable influence on fatigue endurance [2,11,12,13,14,15,16]. 

Even accounting for all above factors, the fatigue tests of FSWed aluminum alloys are often characterized by significant experimental scattering and often show even contradictory results [2]. This effect was probably associated with a diverse character of microstructural changes occurring in different aluminum alloy grades during FSW. In the heat-treatable aluminum alloys, the microstructural evolution is well-accepted to be dominated by dissolution and/or coarsening of strengthening dispersoids which normally leads to material softening [2]. In the non-heat treatable alloys, the structural response is dictated by the recrystallization process [1,2]. In this case, the hardening/softening outcome of FSW depends on the initial material condition. In the cast materials, the recrystallization-induced grain refinement usually provides a strengthening effect. On the other hand, the wrought alloys typically exhibit material softening due to the elimination of dislocation density. 

The present work was undertaken in an attempt to elucidate the common trends in fatigue behavior of FSWed aluminum alloys. To this end, several different alloy grades belonging to both the heat-treatable and non-heat-treatable types in both the cast and wrought temper conditions were employed as program materials. 

## 2. Materials and Methods

### 2.1. Program Materials

To provide a broad view on fatigue behavior aluminum alloy grades, both non-heat treatable Al-Mg-Sc alloy and the heat-treatable commercial 6061 alloy were examined in the present study. The chemical composition of the materials measured by optical emission spectrometry is listed in Table 1. In both cases, the alloys were produced by semi-continuous casting followed by homogenization treatment at 360 °C (Al-Mg-Sc alloy) or 380 °C (6061 alloy) for 12 h. In the Al-Mg-Sc alloy, the obtained material condition was denoted as cast material.

The homogenized ingots were then either rolled (Al-Mg-Sc alloy) or extruded (6061 alloy) to 75% of area reduction at the homogenization temperature. In the Al-Mg-Sc alloy, the produced material condition was referred as hot-rolled material. 

To obtain the peak-hardened condition in the 6061 alloy, the extruded material was subjected to T6 tempering treatment, i.e., solutionized at 550 °C for 1 h, water quenched, and then artificially aged at 160 °C for 8 h. The produced material was denoted throughout as 6061 alloy. 

Further details of the material manufacturing (as well as other experimental procedures) have been described elsewhere [18,19]. The characteristic microstructures of the three above material conditions are summarized in supplementary Figures S1–S3.

### 2.2. FSW Process

In all cases, FSW was conducted using an AccuStir 1004 FSW machine (General Tool Company, Cincinnati, OH, USA). To avoid a formation of the “kissing bond defect”, a double-side FSW was employed. To this end, two sequent FSW passes in mutually opposite directions were applied on the upper and bottom sheet surfaces. In each case, particular welding conditions were selected based on prior experiments [20,21]. The principal directions of FSW geometry were denoted as welding direction (WD), transverse direction (TD), and normal direction (ND). 

In Al-Mg-Sc alloy, the thickness of the welding sheets was 10 mm, and FSW was performed at the spindle (rotation-) rate of 500 rpm and the feed rate of 150 mm/min. The welding tool consisted of a shoulder having a diameter of 16 mm and a threaded probe of 6 mm in length and tapered from 6 mm at the tool shoulder to 4.8 mm at the probe tip. 

In 6061 alloy, 3-mm-thickness welding sheets were used. FSW was conducted at the spindle rate of 1100 rpm and the feed rate of 760 mm/min. The welding tool consisted of a shoulder of 12.5 mm in diameter and an M5 cylindrical probe of 1.9 in length. To recover mechanical properties of the produced weldments, these were artificially aged at 160 °C for 8 h prior to microstructural observations and mechanical tests. 

In all cases, the particular welding variables for each material condition were selected on the basis of the authors’ previous experience in tailoring of mechanical properties of the welded joints for *static* loading conditions.

### 2.3. Microstructure Characterization

In all cases, microstructural examinations were conducted by using optical microscopy, electron backscatter diffraction (EBSD), and transmission electron microscopy (TEM). For optical microscopy, the samples were prepared by mechanical polishing in conventional fashion, followed by the final etching in Keller’s reagent. A final surface finish for EBSD and TEM was obtained by electro-polishing in a solution of 25% nitric acid in ethanol. 

EBSD analysis was performed using a FEI Quanta 600 field-emission-gun scanning electron microscope (FEG-SEM) (Thermo Fisher Scientific, Waltham, MA, USA) equipped with a TSL OIM^TM^ EBSD system (EDAX, Mahwah, NJ, USA). TEM observations were conducted with a JEM-2100EX TEM (JEOL Ltd., Akishima, Japan).

### 2.4. Fatigue Tests

To evaluate fatigue performance of the welded joints, dog-bone-shaped specimens were cut perpendicular to the WD by electrical-discharge machining (EDM). In Al-Mg-Sc alloy, the samples were machined from the weld mid-thickness and had a gauge section of 14 mm in length, 7 mm in width, and 3 mm in thickness. In 6061 alloy, the gauge section of the specimens was of 30 mm in length, 8 mm in width, and 3 mm in thickness. In greater detail, the design of the fatigue specimens is given in supplementary Figure S4. In all cases, the samples were centered at the weld centerline and included all characteristic microstructural zones of FSW. For comparative purposes, appropriate specimens were also prepared from all base material conditions. 

To achieve a uniform thickness and prevent a potential influence of surface defects on fatigue properties, the specimens were mechanically polished to a final 2400 grit size SiC emery paper. Importantly, the lateral surfaces of the specimens remained unpolished and thus retained the EDM-induced recast layer with relatively high roughness. 

The fatigue tests were conducted using an Instron 8801 servo-hydraulic testing system under load control mode and at ambient temperature. A sinusoidal load-time function with a frequency of 25 Hz (in Al-Mg-Sc alloy) or 50 Hz (in 6061 alloy) and a maximum-to-minimum load ratio R = 0.1 was used. The total statistics of the fatigue tests is given in Table 2.

Fracture surface of the failed specimens was studied with FEI Quanta 600 field-emission-gun scanning electron microscope (FEG-SEM).

### 2.5. Static Mechanical Behavior

For the aid of comparison, microhardness measurements and static transverse tensile tests were conducted. Vickers microhardness measurements were carried out by applying a load of 200 g. The tensile tests to failure were performed at an ambient temperature and a nominal strain rate of ~10^−3^ s^−1^. At least two tensile specimens were tested for each material condition.

## 3. Results

### 3.1. Weld Structure

**Figure 1:**
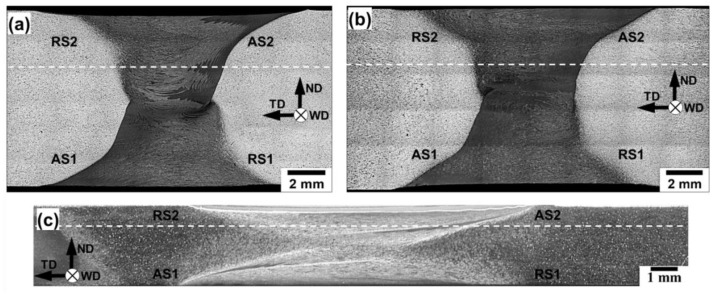
Optical macrographs of the weld cross-sections: (**a**) Al-Mg-Sc alloy in the initial cast condition, (**b**) Al-Mg-Sc alloy in the initial hot-rolled condition, and (**c**) 6061 aluminum alloy. WD, ND, and TD are welding direction, normal direction, and transverse direction, respectively. AS and RS are advancing side and retreating side, respectively; digits indicate a number of friction-stir welding (FSW) pass. Dotted lines show microhardness profiles.

Low-magnification optical images of the weld cross-sections are presented in Figure 1. In all cases, the distinct stir zones with a clear overlapping between two sequential FSW passes are seen. It is evident that all produced welds contain no macro-scale flaws including the “kissing bond” defect.

**Figure 2:**
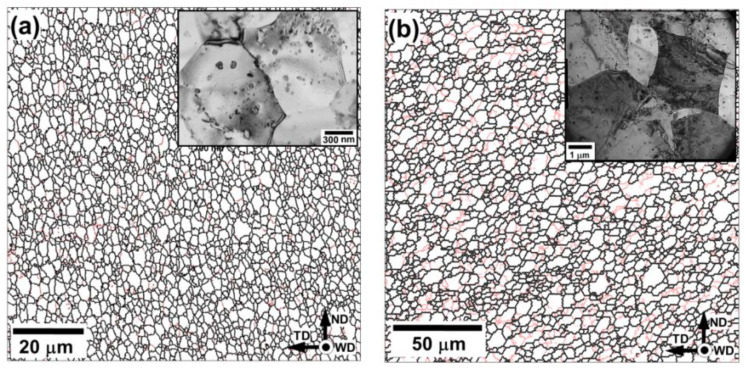
Typical electron backscatter diffraction (EBSD) grain-boundary maps taken from the stir zone center of friction-stir welded (**a**) Al-Mg-Sc alloy and (**b**) 6061 alloy. Transmission electron microscopy (TEM) images of the relevant microstructures are given in the top right corners of the maps. In the EBSD maps, low-angle boundaries (LABs) and high-angle boundaries (HABs) are depicted as red and black lines, respectively. In (**a**), the microstructure of the hot-rolled material condition is shown.

Typical microstructures evolved in the stir zone are summarized in Figure 2. In any case, they were comprised by fine, nearly-equiaxed grains containing low dislocation density. The mean grain size measured by the conventional intercept method was found to be ~1 µm in Al-Mg-Sc alloy and ~5 µm in 6061 alloy. Thus, FSW resulted in considerable grain refinement. These observations were the line with typical FSW literature [1,2] being indicative of a dynamic recrystallization presumably occurred in the stir zone.

A considerable fraction of relatively coarse second-phase particles was also worthy of remark (TEM micrographs in the top right corner of Figure 2). As shown in the previous works [18,19], this finding reflected an essential coarsening (and even partial dissolution) of the secondary particles induced by the weld thermal cycle. 

### 3.2. Preliminary Analysis of Mechanical Properties

**Figure 3:**
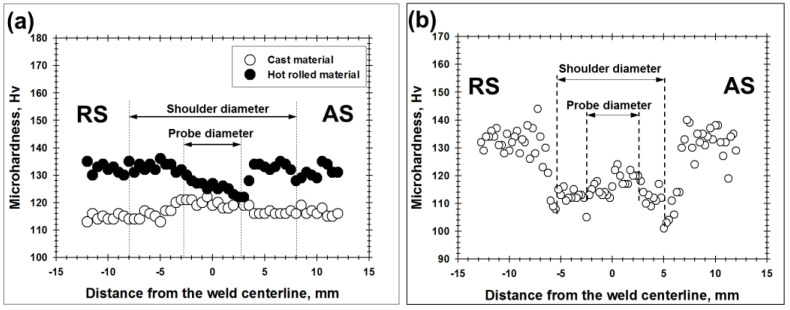
Microhardness profiles measured along the dotted lines shown in Figure 1 in (**a**) Al-Mg-Sc alloy and (**b**) 6061 alloy. For clarity, the probe- and shoulder diameters are indicated. AS and RS are advancing side and retreating side, respectively.

**Figure 4:**
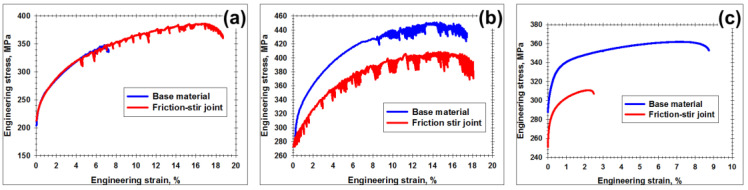
Deformation diagrams comparing tensile behavior of base material and friction-stir joints in (**a**) Al-Mg-Sc alloy with the initial cast condition, (**b**) Al-Mg-Sc alloy with the initial hot-rolled condition, and (**c**) 6061 alloy.

For preliminary evaluation of mechanical properties of the welded joints, microhardness profiles were measured and transverse tensile tests were conducted. The obtained results were summarized in Figure 3 and Figure 4 and Table 3 and Table 4. 

In Al-Mg-Sc alloy in the initial cast condition, FSW gave rise to a subtle material hardening in the stir zone (Figure 3a). This was presumably due to the substantial grain refinement in this microstructural region (Figure 2a). As a result, the welded joints exhibited a tensile behavior broadly similar to the parent material with the failure occurring in the base material zone (Figure 4a and Table 3). 

In contrast, FSW of Al-Mg-Sc alloy in initial hot-rolled condition resulted in essential material softening (Figure 3b). As demonstrated by Malopheyev et al. [20], this effect was attributable to the elimination of the original work-hardened substructure due to the recrystallization occurred in the stir zone. Accordingly, the subsequent transverse tensile tests of the welded joints led to the strain localization in the softened stir zone which degraded the global strength characteristics (Figure 4b and Table 3). 

In the heat-treatable 6061 alloy, FSW also exerted a pronounced softening effect (Figure 3b). As shown in the previous work [21], this was due to the coarsening of strengthening precipitates induced by the weld thermal cycle. Hence, the subsequent transverse tensile tests also resulted in the strain localization, and thus mechanical properties of the welded joints were comparatively low (Figure 4c, Table 4).

### 3.3. Fatigue Diagrams

**Figure 5:**
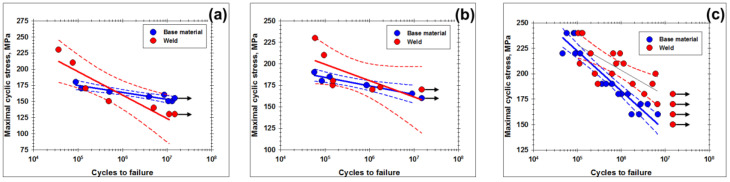
Fatigue lifetime versus maximal cyclic stress per cycle for base material and friction-stir joints in (**a**) Al-Mg-Sc alloy with the initial cast condition, (**b**) Al-Mg-Sc alloy with the initial hot-rolling condition, and (**c**) 6061 alloy. In the figures, solid lines represent median curves, whereas dotted lines show 95% confidence bands. Arrows indicate run-out tests.

The effect of cyclic loading on fatigue life of the base materials and the welded joints are shown in Figure 5 and Table 5 and Table 6. To provide additional insight to the results, the data shown in Figure 5 were statistically analyzed. For this purpose, the run-out tests were excluded from consideration, whereas the remaining results were linearly fitted.

Quite expectedly, a reduction in cycles stress extended the fatigue lifetime but no clear saturation or a “fatigue limit” was revealed in the fatigue diagrams (Figure 5). This finding was in a close agreement with a typical behavior of fatigued aluminum alloys. Of particular interest was the observation that the welded joints exhibited the fatigue strength comparable with that of the base materials in all investigated alloy grades and the initial temper conditions (Figure 5).

Remarkably, the fatigue behavior of the welded Al-Mg-Sc alloy was characterized by significant experimental scattering. Though the origin of this effect is not completely clear, one of the possible explanations may be due to the limited number of tested specimens (Table 2). To clarify this issue, additional fatigue tests are needed. 

Another remarkable issue was the failure location of the welded specimens. As follows from Table 5 and Table 6, at relatively low fatigue stresses (below ~0.75 fraction of the static yield strength), all joints failed in the base material zone. At higher cyclic stresses, however, the fatigue fracture may also occur in the weld zone.

### 3.4. Fatigue Fracture

**Figure 6:**
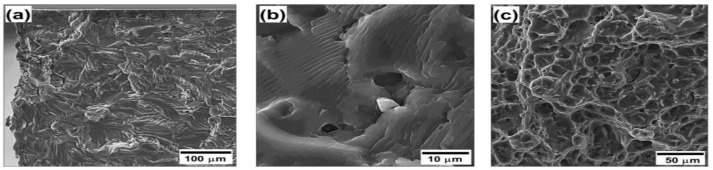
SEM micrographs illustrating fracture surface of friction-stir welded Al-Mg-Sc alloy in the cast initial condition fatigued at maximal cyclic stress of 140 MPa: (**a**) crack nucleation, (**b**) discontinuous crack propagation, and (**c**) catastrophic failure.

In order to provide an additional insight into fatigue behavior of the welded joints, fracture surfaces of the fatigued specimens was examined with typical results being shown in Figure 6. In all cases, three characteristic fracture zones (representing three typical stages of the fatigue failure) could be defined: (i) crack initiation, (ii) crack propagation, and (iii) catastrophic failure [22,23]. 

In most cases, the fatigue crack initiated at the lateral surface of the fatigued specimens (Figure 6a). This observation is thought to be associated with relatively low quality of such surfaces produced by EDM, as mentioned in Section 2.4. In stage II, the fracture surface was dominated by the fatigue striations (Figure 6b) which are usually attributed to a discontinuous character of the crack propagation (from cycle to cycle). The stage III was characterized by dimpled appearance (Figure 6c) indicating the ductile fracture mechanism associated with nucleation and coalescence of voids. 

It is important to point out that the fracture surface in all cases was dominated by the stage III (not shown). This perhaps implies a relatively low resistance to a propagation of the fatigue crack. Striation patterns in Figure 6b support this conclusion. If so, the fatigue performance of the studied welds was probably controlled by the crack nucleation event.

## 4. Discussion

### 4.1. Broad Aspects of Fatigue Performance

As mentioned above, the fatigue performance of friction-stir welded joints is believed to be governed by four primary factors including (i) welding defects, (ii) surface quality, (iii) residual stress, and (iv) microstructure [2]. Since the welds examined in the present study contained no internal flaws and were mechanically polished to remove the tool marks, the fatigue cracking was perhaps dictated by two latter factors. 

The residual stresses generated in the weld zone are normally tensile in nature and thus they should promote fatigue cracking. On the other hand, the considerable grain refinement in the stir zone, induced during FSW (Figure 2), is beneficial for fatigue resistance. This effect is usually attributed to the suppression of slip banding in the fine-grained materials which often serve as a precursor for the fatigue crack initiation [24,25,26]. It seems, therefore, that the fatigue behavior of the studied welds resulted from the competitive influence of two above inherent characteristics of the FSW process.

Given the revealed dependence of the failure location from the magnitude of cyclic stress (Table 5 and Table 6), a difference in the mechanism of the fatigue cracking was suggested. Accordingly, the fatigue behavior at low-and high fatigues stresses was considered separately in the following two sections.

### 4.2. Fatigue Behavior at Low Cyclic Stress

As shown in Table 5 and Table 6, the welds fatigued at relatively low cyclic stress (with the peak magnitude below ~0.74 fraction of the static yield strength) typically failed in the base material zone. It is worth noting that the base material region of the welded joints is normally characterized by compressive residual stress [2] and was comprised by the relatively coarse-grained microstructure (supplementary Figures S1–S3). It could be concluded therefore that the adverse influence of the microstructural factor on the fatigue cracking in this case was stronger that the positive effect of the compressive stress. 

**Figure 7:**
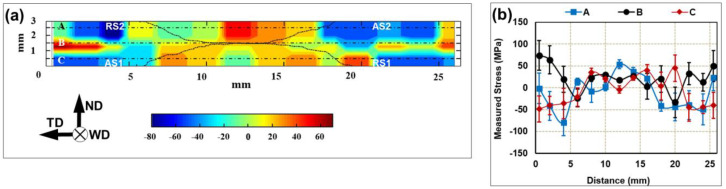
(**a**) Distribution of residual stress on the weld cross-section and (**b**) profiles of the residual stress measured along the lines indicated in (**a**). For clarity, the stir zone borderlines are outlined in (**a**) as dotted lines.

Hence, it seems that the nucleation of the fatigue crack at low cyclic stresses was governed by the microstructure rather than the residual stresses. Attempting to comprehend this result, a magnitude of the residual stresses was measured in the welded 6061 alloy by x-ray diffraction (XRD) technique using a PROTO-LXRD diffractometer (Proto Manufacturing Ltd., Oldcastle, ON, Canada) and the sin^2^ψ approach. A cobalt target and accelerated voltage of 25 kV were used. The stress were calculated from the strains of the {311} Bragg reflection at 148.9. To examine a distribution of the residual stress on the weld cross-section, the measurements were conducted on a rectangular grid with a step size of 1 mm. For each point, 10 measurements with 1-s exposure time were performed. Further experimental details are given in Ref. [19]. The obtained results were shown in Figure 7. From this figure, it is seen that the peak residual stress did not exceed +60 MPa in the stir zone and −80 MPa in the base material, thus constituting only ~25% of the static yield strength (Table 4). The relatively low residual stresses revealed in the present study are in the line with literature data [2] and are likely attributable to the solid-state nature of the FSW process, implying the comparatively low heat input. Moreover, an additional factor promoting a formation of relatively low residual stress, may be a double-sided FSW mode used in the present study, as mentioned in Section 2.2. In the case, the heat-input generated during second FSW pass could partially relieve the residual stresses in the weld zone. 

Thus, considering the relatively low residual stress but drastic grain refinement, both attributable to FSW, it is expected that the fatigue crack at low cyclic stresses should typically nucleate in the coarse-grained base material region. Accordingly, the welded joints should exhibit the fatigue strength comparable to that of the parent material, as indeed observed in the present study (Figure 5).

### 4.3. Fatigue Behavior at High Cyclic Stress

The welds fatigued at relatively high cyclic stress (with the peak magnitude ≥0.74 fraction of the static yield strength) often failed either in stir zone (Table 5) or the heat-affected zone (Table 6). In this case, the summary stress (i.e., the cyclic stress plus the residual stress) could exceed the material yield strength; thus, the fatigued specimen may experience a plastic strain before nucleation of a fatigue crack.

**Figure 8:**
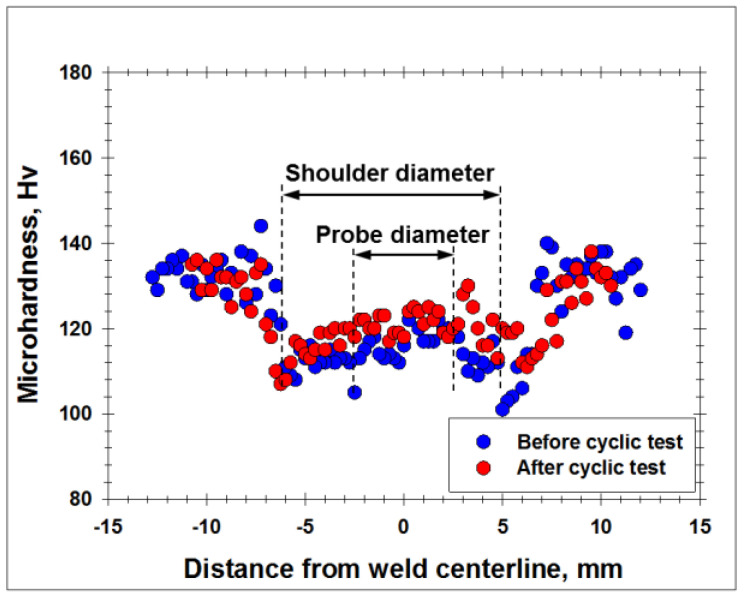
Effect of the fatigue tests at high cyclic stress on microhardness profile measured across the welded joint of 6061 alloy. See Section 4.3 for details. Note: The data were taken from the specimen fatigued at the maximal stress of 240 MPa.

To examine this suggestion, appropriate microhardness profiles were measured across the welded specimen before- and after the fatigue tests, and the typical result is shown in Figure 8. A measurable strain hardening seen in the weld zone appears to confirm the above suggestion. It may be suggested, therefore, that the fatigue cracking at relatively high cyclic stresses should be induced by the preceding plastic strain and thus should occur in the softest microstructural region of the welded joint. This conclusion agrees well with the recent work by Ma et al. [27], in which a measurable strain hardening effect has been found in low-cycle fatigued aluminum alloys.

In the present work, the welding conditions were carefully tailored in order to minimize the softening effect in the wrought Al-Mg-Sc alloy and the heat-treatable 6061 alloy (Figure 3). Accordingly, the welded specimens demonstrated the fatigue strength comparable to that of the base material (Figure 5). It is well known, however, that an improper combination of FSW variables may essentially deteriorate the static weld strength [21]. In this case, a substantial reduction of the fatigue endurance is expected.

## 5. Summary

Despite a vast volume of experimental data on fatigue behavior of FSWed aluminum alloys existing in the scientific literature, the generic characteristics of this phenomenon are still unclear. The present study was undertaken to shed some light on this issue. To this end, (i) defect-free welds were obtained in various alloy grades and initial temper conditions, (ii) the fatigue specimens were carefully machined to remove the characteristic tool marks and the “kissing bond” defects, and (iii) the data analysis was based on the presumption of the dominant role of the FSW-induced residual stress and microstructure in fatigue cracking. 

Since FSW typically results in tensile residual stresses in the weld zone (which are harmful for the fatigue life) and considerable grain refinement (which is beneficial for the fatigue strength), it was suggested that the fatigue behavior of the FSW joints was governed by the competitive influence of these two factors. It was shown that the residual stress constituted only ~25% of the static yield strength, thus being relatively low. On the other hand, the mean grain size in the stir zone was found to vary from 1 to 5 μm, thus indicating a pronounced character of the grain refinement effect. 

In a view of the above circumstances, the fatigue performance at relatively low cyclic stress was deduced to be dictated by the microstructural factor. Accordingly, the fatigue crack typically nucleated in relatively coarse-grained base material zone; thus, the fatigue strength of the welded joints was comparable to that of the parent metal. 

At relatively high fatigue stress, however, the summary (i.e., the cyclic-plus residual-) stress may exceed the material yield strength and thus the fatigue cracking should result from the preceding macro-scale plastic deformation. Accordingly, the fatigue failure should occur in the softest microstructural region; thus, the fatigue strength of the welded joint should be inferior to that of the original material.

## Figures and Tables

**Table 1:** Measured chemical composition of program materials (wt.%).

Alloy	Al	Mg	Mn	Si	Fe	Cu	Sc	Zr	Cr	Zn
Al-Mg-Sc	Bal.	6.0	0.35	–	–	–	0.2	0.08	0.07	–
AA6061		0.88	0.12	0.66	0.72	0.26	–	–	0.12	0.09

**Table 2:** Statistics of fatigue tests.

Fatigue Stress, MPa		Number of Tested Specimens					
Amplitude	Max Magnitude	Al-Mg-Sc Alloy, Initial Cast Condition		Al-Mg-Sc Alloy, Initial Hot-Rolled Condition		6061 Alloy	
		Base Metal	Weld	Base Metal	Weld	Base Metal	Weld
58.5	130	–	2	–	–	–	–
63	140		1				
67.5	150	2	1			2	5
69.8	155	1	–			–	–
70.7	157	1					
72	160	1				3	3
74.3	165	1					
76.5	170	1	1		2	2	4
77.6	172.5	–	–		1	–	–
78.8	175				1		
81	180	1			1	3	2
84.5	190	–		2	–	3	3
86.4	192			1		–	–
87.8	195			1			
90	200			1			3
94.5	210		1	1	1		3
99	220			1		3	3
103.5	230		1	1	1		
108	240					3	3

**Table 3:** Tensile properties of Al-Mg-Sc alloy.

Material Condition		Yield Strength, MPa	Ultimate Tensile Strength, MPa	Elongation to Failure, %	Fracture Location
Cast	Base material	232 ± 12	348 ± 11	7 ± 1	
	Welded joint	233 ± 17	386 ± 20	19 ± 2	Base material
Hot-rolled	Base material	288 ± 8	451 ± 10	17 ± 1	
	Welded joint	285 ± 15	409 ± 12	18 ± 2	Stir zone

**Table 4:** Tensile properties of 6061 alloy.

Material Condition	Yield Strength, MPa	Ultimate Tensile Strength, MPa	Elongation to Failure, %	Fracture Location
Base material	316 ± 3	362 ± 1	9 ± 1	
Welded joint	283 ± 7	311 ± 12	3 ± 1	Heat-affected zone

**Table 5:** Results of fatigue tests of Al-Mg-Sc alloy.

Base Metal			Welded Metal			
Max Cyclic Stress		Cycles to Failure	Max Cyclic Stress		Cycles to Failure	Failure Location
Magnitude, MPa	Fraction of Yield Stress		Magnitude, MPa	Fraction of Yield Stress		
Cast material condition						
150	0.65	12,975,503	130	0.56	Run-out test	
		10,821,207			11,130,650	Base metal
155	0.67	Run-out test	140	0.60	5,061,109	
157	0.68	3,950,713	150	0.64	493,988	
160	0.69	8,616,097	170	0.73	147,339	
165	0.71	507,973	210	0.90	76,486	
170	0.73	119,353	230	0.99	36,727	
180	0.78	89,452				
Hot rolled material condition						
160	0.56	Run-out test	170	0.60	Run-out test	
165	0.57	9,112,783			11,660,890	Base metal
170	0.59	Run-out test	172.5	0.61	1,750,082	
175	0.61	863,163	175	0.61	149,002	
180	0.63	84,993	180	0.63	150,880	
185	0.64	126,688	210	0.74	95,290	
190	0.66	57,240	230	0.81	59,479	Stir zone

**Table 6:** Results of fatigue tests of 6061 alloy.

Base Material			Welded Metal			
Max Cyclic Stress		Cycles to Failure	Max Cyclic Stress		Cycles to Failure	Failure Location
Magnitude, MPa	Fraction of Yield Stress		Magnitude, MPa	Fraction of Yield Stress		
150	0.47	2 Run-out tests	150	0.53	5 Run-out tests	
160	0.51	2,538,856	160	0.57	3 Run-out tests	
		6,764,240	170	0.60	2 Run-out tests	
		1,704,748			6,717,387	Base metal
170	0.54	2,763,392	180	0.64	Run-out test	
		4,020,179			3,360,543	Base metal
180	0.57	1,006,680	190	0.67	292,961	
		1,417,184			5,183,806	
		861,513			1,829,003	
190	0.60	620,615	200	0.71	619,010	
		364,845			6,029,954	
		452,393			248,991	
220	0.70	90,369	210	0.74	771,268	HAZ
		115,993			1,145,951	
		46,472			112,994	Base metal
240	0.76	83,048	220	0.78	202,805	HAZ
		87,718			943,967	
		57,046			661,812	
			240	0.85	125,552	Base metal
					104,777	
					129,689	HAZ

## Supplementary Materials

The following are available online at https://www.mdpi.com/1996-1944/13/19/4246/s1, Figure S1: Initial microstructure of the cast Al-Mg-Sc alloy: optical micrograph (a), EBSD grain-boundary map (b) and TEM images (c, d). In (b), low-and high-angle boundaries are depicted as red and black lines, respectively, Figure S2, Initial microstructure of the hot-rolled Al-Mg-Sc alloy: optical micrograph (a), EBSD grain-boundary map (b) and TEM images (c), (d). In (b), low- and high-angle boundaries are depicted as red and black lines, respectively, Figure S3, Initial microstructure of 6061 alloy: (a) EBSD grain-boundary map and (b) TEM image. ED is extrusion direction. In (a), low-and high-angle boundaries are depicted as red and black lines, respectively, Figure S4, Design of the fatigue specimens machined from the welded sheets of Al-Mg-Sc alloy (a) and 6061 alloy (b). In all cases, units are mm. Not in scale

## References

[1] Mishra R.S., Ma Z.Y. (2005). Friction stir welding and processing. Mater. Sci. Eng. R.

[2] Threadgill P.L., Leonard A.J., Shercliff H.R., Withers P.J. (2009). Friction stir welding of aluminum alloys. Int. Mater. Rev..

[3] Texier D., Atmani F., Bocher P., Nadeau F., Chen J., Zedan Y., Vanderesse N., Demers V. (2018). Fatigue performance of FSW and GMAW aluminum alloys welded joints: Competition between microstructural and structural-contact-fretting crack initiation. Int. J. Fatigue.

[4] Ericsson M., Sandstrom R. (2003). Influence of welding speed on the fatigue of friction stir welds, and comparison with MIG and TIG. Int. J. Fatigue.

[5] Di S.S., Yang X.Q., Luan G.H., Jian B. (2006). Comparative study on fatigue properties between AA2024-T4 friction stir welds and base materials. Mater. Sci. Eng. A.

[6] Uematsu Y., Tokaji K., Shibata H., Tozaki Y., Ohmune T. (2009). Fatigue behavior of friction stir welds without neither welding flash nor flaw in several aluminum alloys. Int. J. Fatigue.

[7] Zhou C., Yang X., Luan G. (2006). Effect of root flaws on the fatigue property of friction stir welds in 2024-T3 aluminum alloys. Mater. Sci. Eng. A.

[8] Oosterkamp A., Oosterkamp L.D., Nordeide A. (2004). “Kissing bond” phenomena in solid-state welds of aluminum alloys. Weld. J..

[9] Dickerson T.L., Przydatek J. (2003). Fatigue of friction stir welds in aluminum alloys that contain root flaws. Int. J. Fatigue.

[10] Lomolino S., Tovo R., Dos Santos J. (2005). On fatigue behavior and design curves of friction stir butt-welded Al alloys. Int. J. Fatigue.

[11] Li W.Y., Li N., Yang X.W., Feng Y., Vairis A. (2017). Impact of cold spraying on microstructure and mechanical properties of optimized friction stir welded AA2024-T3 joint. Mater. Sci. Eng. A.

[12] Hatamleh O. (2009). A comprehensive investigation on the effect of laser and shot peening on fatigue crack growth in friction stir welded AA 2195 joints. Int. J. Fatigue.

[13] Fratini L., Pasta S., Reynolds A.P. (2009). Fatigue crack growth in 2024-T351 friction stir welded joints: Longitudinal residual stress and microstructural effects. Int. J. Fatigue.

[14] Hatamleh O., Lyons J., Forman R. (2007). Laser and shot peening effects on fatigue crack growth in friction stir welded 7075-T7351 aluminum alloy joints. Int. J. Fatigue.

[15] John R., Jata K.V., Sadananda K. (2003). Residual stress effects on near-threshold fatigue crack growth in friction stir welds in aerospace alloys. Int. J. Fatigue.

[16] Bussu G., Irving P.E. (2002). The role of residual stress and heat affected zone properties on fatigue crack propagation in friction stir welded 2024-T351 aluminum joints. Int. J. Fatigue.

[17] Sato Y.S., Takauchi H., Park S.H.C., Kokawa H. (2005). Characteristics of the kissing-bond in friction stir welded Al alloy 1050. Mater. Sci. Eng. A.

[18] Zhemchuzhnikova D., Mironov S., Kaibyshev R. (2017). Fatigue performance of friction-stir-welded Al-Mg-Sc alloy. Metall. Mater. Trans. A.

[19] Vysotskiy I., Malopheyev S., Rahimi S., Mironov S., Kaibyshev R. (2019). Unusual fatigue beauvor of friction-stir welded Al-Mg-Si alloy. Mater. Sci. Eng. A.

[20] Malopheyev S., Kulitskiy V., Mironov S., Zhemchuzhnikova D., Kaibyshev R. (2014). Friction-stir welding of an Al-Mg-Sc-Zr alloy in as-fabricated and work-hardened conditions. Mater. Sci. Eng. A.

[21] Malopheyev S., Vysotskiy I., Kulitskiy V., Mironov S., Kaibyshev R. (2016). Optimization of processing-microstructure-properties relationship in friction-stir welded 6061-T6 aluminum alloy. Mater. Sci. Eng. A.

[22] ASM International (1987). ASM Handbook: Fractography.

[23] ASM International (1996). ASM Handbook: Fracture and Fatigue.

[24] Vinogradov A., Washikita A., Kitagawa K., Kopylov V.I. (2003). Fatigue life of fine-grain Al-Mg-Sc alloys produced by equal-channel angular pressing. Mater. Sci. Eng. A.

[25] Mughrabi H., Hoppel H.W. (2010). Cyclic deformation and fatigue properties of very fine grained metals and alloys. Int. J. Fatigue.

[26] Estrin Y., Vinogradov A. (2010). Fatigue behavior of light alloys with ultrafine grain structure produced by severe plastic deformation: An overview. Int. J. Fatigue.

[27] Ma Z.Y., Feng A.H., Chen D.L., Shen J. (2018). Recent Advances in Friction Stir Welding/Processing of Aluminum Alloys: Microstructural Evolution and Mechanical Properties. Crit. Rev. Solid State Mater. Sci..

