# Supplementary material for: On the Fatigue Performance of Friction-Stir Welded Aluminum Alloys
Source: Materials (Basel). 2020 Sep 23;13(19):4246. doi: 10.3390/ma13194246 (PMC7579519; doi:10.3390/ma13194246)
Supplement: Supplementary file 1 [file materials-13-04246-s001.pdf]

On the Fatigue Performance of Friction-Stir Welded Aluminum Alloys

Sergey Malopheyev, Igor Vysotskiy, Daria Zhemchuzhnikova, Sergey Mironov * and Rustam Kaibyshev

Laboratory of Mechanical Properties of Nanoscale Materials and Superalloys,
Belgorod National Research University, 308015 Belgorod, Russia; malofeev@bsu.edu.ru (S.M.);
isotsky@bsu.edu.ru (I.V.); zhemchuzhnikova@bsu.edu.ru (D.Z.); rustam_kaibyshev@bsu.edu.ru (R.K.)
* Correspondence: mironov@bsu.edu.ru; Tel.: +7-4722-585455

Received: 19 August 2020; Accepted: 21 September 2020; Published: date

Supplementary Materials

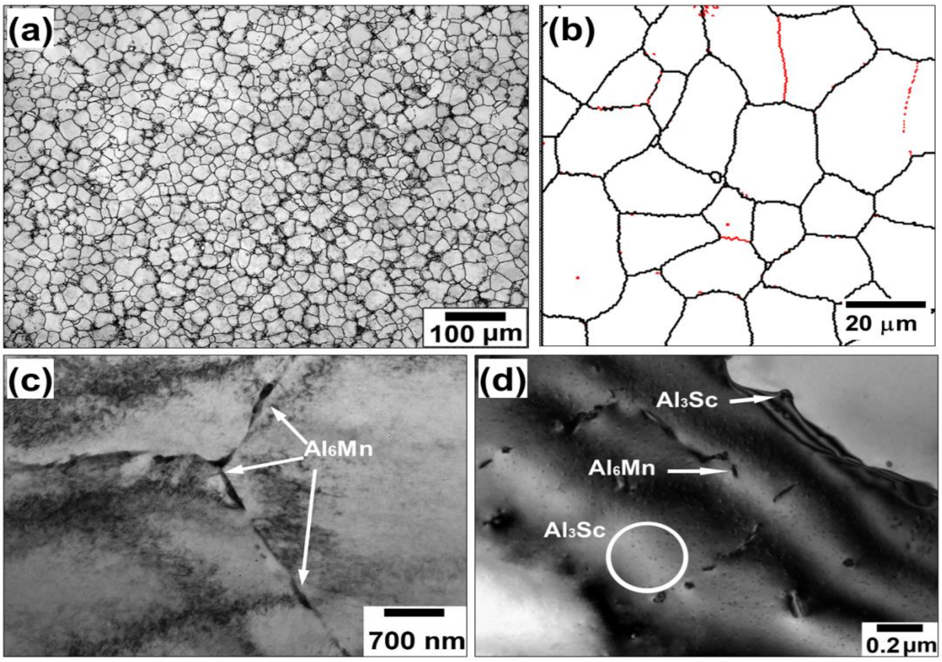

Figure S1. Initial microstructure of the cast Al-Mg-Sc alloy: optical micrograph (**a**), EBSD grain-boundary map (**b**) and TEM images (**c, d**). In (b), low-and high-angle boundaries are depicted as red and black lines, respectively.

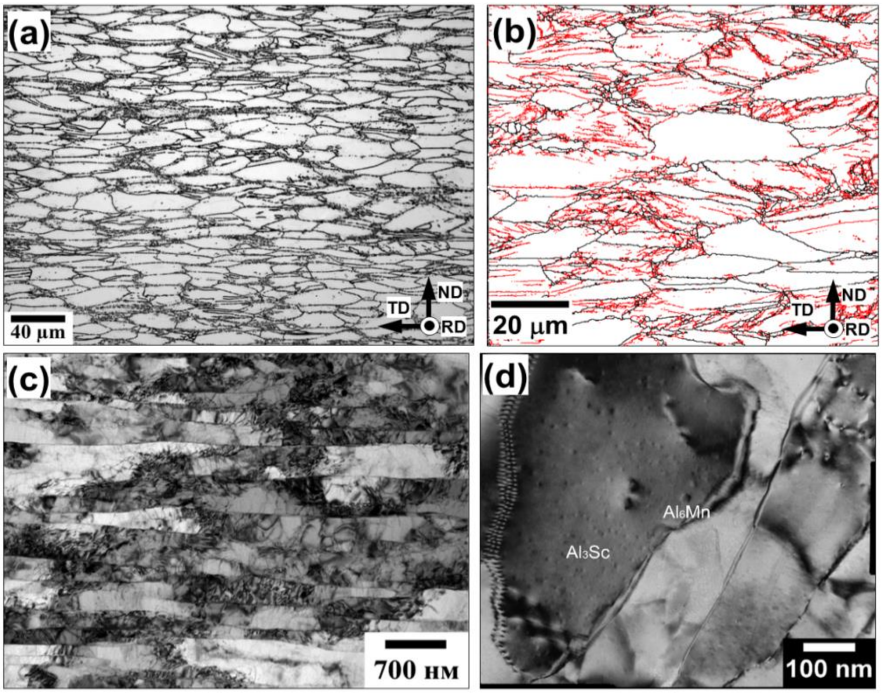

Figure S2. Initial microstructure of the hot-rolled Al-Mg-Sc alloy: optical micrograph (**a**), EBSD grain-boundary map (**b**) and TEM images (**c**), **(d**). In (**b**), low- and high-angle boundaries are depicted as red and black lines, respectively.

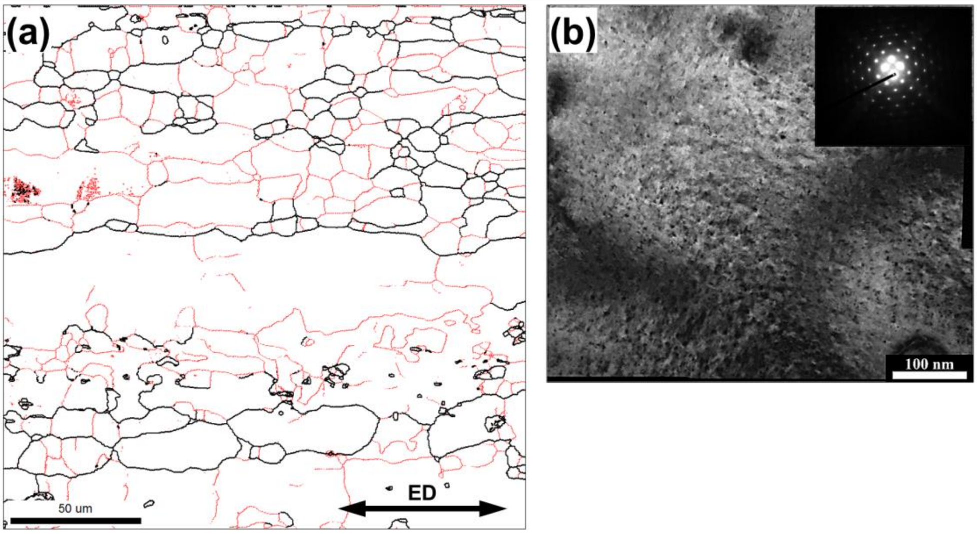

Figure S3. Initial microstructure of 6061 alloy: (**a**) EBSD grain-boundary map and (**b**) TEM image. ED is extrusion direction. In (**a**), low-and high-angle boundaries are depicted as red and black lines, respectively.

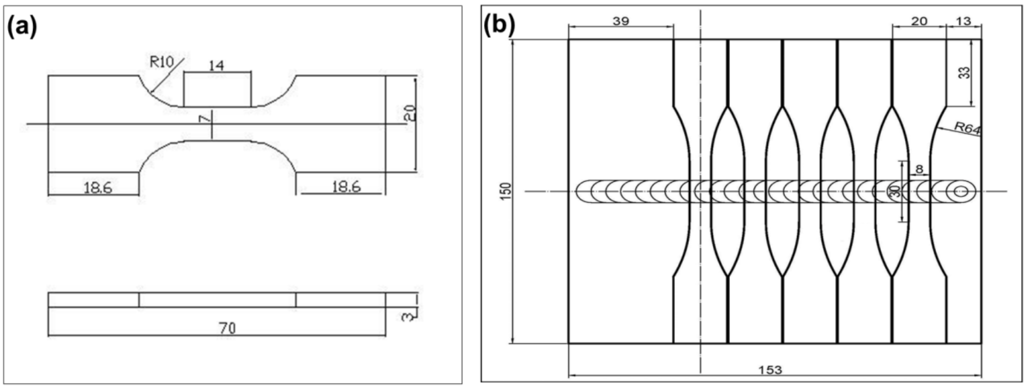

Figure S4. Design of the fatigue specimens machined from the welded sheets of Al-Mg-Sc alloy (**a**) and 6061 alloy (**b**). In all cases, units are mm. Not in scale.
